# Congenital hyperinsulinsim: case report and review of literature

**DOI:** 10.11604/pamj.2020.35.53.16604

**Published:** 2020-02-24

**Authors:** Brahim El Hasbaoui, Abdelhkim Elyajouri, Rachid Abilkassem, Aomar Agadr

**Affiliations:** 1 Department of pediatrics, Military Teaching Hospital Mohammed V, Faculty of Medicine and Pharmacy, University Mohammed V, Rabat, Morocco

**Keywords:** Congenital hyperinsulinism, neonatal hypoglycemia, insulin, gene mutations

## Abstract

Neonatal hypoglycemia (NH) is one of the most common abnormalities encountered in the newborn. Hypoglycemia continues to be an important cause of morbidity in neonates and children. Prompt diagnosis and management of the underlying hypoglycemia disorder is critical for preventing brain damage and improving outcomes. Congenital hyperinsulinism (CHI) is the most common and severe cause of persistent hypoglycemia in neonates and children, it represents a group of clinically, genetically and morphologically heterogeneous disorders characterised by dysregulation of insulin secretion from pancreatic β-cells. It is extremely important to recognize this condition early and institute appropriate management to prevent significant brain injury leading to complications like epilepsy, cerebral palsy and neurological impairment. Histologically, CHI is divided mainly into two types focal and diffuse disease. The diffuse form is inherited in an autosomal recessive (or dominant) manner whereas the focal form is sporadic in inheritance and is localized to a small region of the pancreas. Recent discoveries of the genetic causes of CHI have improved our understanding of the pathophysiology, but its management is complex and requires the integration of clinical, biochemical, molecular, and imaging findings to establish the appropriate treatment according to the subtype. Here we present a case of sever congenital hyperinsulinism in a girl admitted for lethargy, irritability and general seizures accompanied with profound hypoglycemia, in spite of aggressive medical treatment, she died because of sever congenital hyperinsulinism diazoxide unresponsive.

## Introduction

Glucose is a source of energy storage in the form of glycogen, fat, and protein. Glucose provides 38mol of Adenosine Triphosphate (ATP) per mol of glucose oxidized. It’s the preferred substrate for the energetic needs of the brain. Cerebral transport of glucose is a carrier mediated facilitated diffusion process that is dependent on blood glucose concentration and is not regulated by insulin. The protection against hypoglycemia is coordinated by the autonomic nervous system and by hormones, which stimulate glucose production through glycogennolysis and gluconeogenesis and limit peripheral glucose utilization.

Knowledge of the homeostatic mechanisms that maintain blood glucose concentration between the relatively narrow ranges of 65-100 mg/dL during fasting is the key for the diagnosis and appropriate management of hypoglycemia. During feeding, absorbed glucose in excess of immediate requirements is stored as glycogen in the liver to be utilized during fasting. Between feeds, normal blood glucose levels are maintained by glycogenolysis (liver) and gluconeogenesis (liver and kidney). Gluconeogenesis is a process by which glucose is produced from non-carbohydrate carbon substrates such as pyruvate, lactate, glucogenic amino acids (especially alanine) and glycerol.

The human fetus receives continuous supply of nutrients through placental circulation. After birth, the newborn infant’s metabolism must adapt to the fast-feed cycle. The hormonal changes (decrease in plasma insulin and increase in glucagon and catecholamines) at birth allow the newborn infant to adapt successfully to interrupted supply of nutrients. Disturbances in this smooth transition can result in neonatal hypoglycemia. Most cases of neonatal hypoglycemia are due to delay of the normal processes of metabolic adaptation after birth and occur in at-risk infants (Table 1) [1, 2]. An underlying metabolic or hormonal etiology should be suspected when the hypoglycemia is of unusual severity or occurs in an otherwise low-risk infant. Some metabolic and hormonal conditions presenting with hypoglycemia in the neonatal period are shown in Table 2 [3, 4 ].

**Table 1:** Transient hypoglycemia causes

Maternal conditions	Neonatal conditions
Pre-gestational or gestational diabetes Medication (β-blockers, oral hypoglycemic agents, intrapartum glucoseadministration)	Prematurity Small for gestational age/ IUGR Large for gestational age Perinatal hypoxia-ischemia Infection Polycythemia Hypothermia Parenteral nutrition Syndromic features, midline defects

**Table 2:** Metabolic and endocrine causes of neonatal hypoglycemia

**Hyperinsulinism**	**Transient** Infant of diabetic mother Perinatal asphyxia Rhesus hemolytic disease Intrauterine growth restriction (IUGR) Beckwith-Wiedemann syndrome **Congenital** ABCC8/ KCNJ11/ GCK/ GDH/ HADH/ HNF4A
**Hypoinsulinemic hypoglycemia**	Activating AKT2 mutations
**Counter-regulatory hormone deficiency**	Growth hormone deficiency Adrenal insufficiency
**Fatty acid oxidation disorders**	Medium chain acyl-CoA dehydrogenase deficiency Long chain acyl-CoA dehydrogenase deficiency Short chain acyl-CoA dehydrogenase deficiency
**Defects in ketone body synthesis/ utilization**	HMG CoA synthase deficiency HMG CoA lyase deficiency
**Carnitine deficiency (primary and secondary)**	Carnitine palmitoyl transferase deficiency (CPT 1 and 2), Carnitine deficiency
**Gluconeogenic disorders**	Fructose-1, 6-bisphosphatase deficiency, Phosphoenolpyruvate carboxykinase (PEPCK) deficiency, Pyruvate carboxylasedeficiency
**Glycogen storage disorders**	Glucose-6-phosphatase deficiency Amylo 1–6 glucosidase deficiency Glycogen synthase deficiency
**Defects in glucose transport**	GLUT 1/2/3 transporters defects
**Other metabolic conditions**	Galactosemia, Fructosemia, Tyrosinemia, Glutaric aciduria type 2, Maple syrup urine disease Propionic acidemia

The definition of neonatal hypoglycemia (NH) is a subject of great controversy [5, 6]. However, it is accepted that NH is defined by a plasma glucose level of less than 30 mg/dL (1.65 mmol/L) in the first 24 hours of life and less than 45 mg/dL (2.5 mmol/L) thereafter.

## Patient and observation

We are reporting a case of a girl with normal perinatal history, she was born to a 32-year-old primigravida mother at 39-week gestation by caesarean section for non-reassuring fetal status. She was born from a consanguineous parent. There was no family history of diabetes or hypoglycaemia either intra-partum drugs administered to the mother (glucose infusions, oral hypoglycaemic agents). No episodes of hypoglycaemia or possibly misdiagnosed as infantile seizures or unexplained deaths were remarked in other family members. Antenatal ultrasound scan showed normal growth with normal Doppler. The Apgar scores were 7 and 10 at 1 and 5min, respectively. She was not dysmorphic, her weight, head circumference and length were within the normal range. No syndromic features or organomegaly/hepatomegaly were remarked. She was admitted after birth to the intensive care unit because of respiratory distress requiring ventilation for 36 h and generalized seizure accompanied with hypoglycemia. Regular feeding every 2h with increased amount of food was sufficient to maintain normoglycemia. The patient was discharged and an out-patient follow-up was instituted without any treatment.

The girl was admitted again at day 40 of age because of irritability, lethargy and generalized seizures accompanied by recurrent episodes of hypoglycemia. Septic markers were unremarkable. The screening tests for inborn error of metabolism were negative. Serum ammonia, lactate cortisol and IGF1 levels were normal. At the time of hypoglycemia (0.9 mmol/L) insulin and C-peptide levels were increased (insulin, 55.9 µU/mL; C-peptide, 2.2 ng/mL), Serum ketones were negative and acyl carnitine levels were low leading to the diagnosis of hyperinsulinism. There was a rise in glucose levels following glucagon administration. Congenital hyperinsulinism was diagnosed through a combination of clinical and laboratory findings, including clinical presentation, hyperinsulinism, low serum ketone bodies, low serum fatty acids with increase of blood sugar levels after glucagon injection. In view of the initial diazoxide unresponsiveness, genetic studies were performed for the baby and the parents.

The treatment was started with increasing glucose infusions (maximum of 22 mg/kg/min) for maintaining normoglycaemia in combination with oral diazoxide (20 mg/kg/day). This measure was not effective and repeated episodes of hypoglycemia were observed 2-3 times a day leading to combined treatment with intramuscular injections of Octreotide (40 mg/kg/day) maintained glycemia within normal range for several days, yet it had to be discontinued because of side effects (persistent vomiting, functional invagination). Episodes of hypoglycemia became more frequent and intravenous glucose requirement increased up to 12 mg/kg/min to maintain normoglycemia. A surgical opinion for possible pancreatectomy was made in light of the high Glucose infusion requirement and initial diazoxide unresponsiveness while continuing with the aggressive medical management, pending genetic study report. Unfortunately, she died at the 6 months of age because of generalized seizures accompanied with profound hypoglycemia.

## Discussion

Congenital HI is a major cause of persistent and recurrent hypoglycaemia in the neonatal and infancy periods. It is characterised by inappropriate and unregulated secretion of insulin from pancreatic β-cells in relation to the blood glucose concentration. Patients with CHI have increased risk of brain injury secondary to the metabolic actions of insulin, which acts by driving glucose into the insulin sensitive tissues (skeletal muscle and adipose tissue) and by inhibiting glucose production by glycolysis and gluconeogenesis [7]. It also inhibits fatty acid release and ketone body synthesis; hence the brain is deprived of both its primary and secondary energy sources (glucose & ketone bodies). CHI is a heterogeneous condition in terms of clinical presentation, histological subgroups and underlying molecular biology. The incidence of CHI can vary from 1 in 35,000-40,000 in the general population to 1 in 2500 in some communities with high rates of consanguinity [8]. The term congenital HI was proposed in 1976 by Stanley and Baker, who outlined the diagnostic criteria for HI: (1) hyperinsulinemia, (2) hypoketonemia, (3) hypo-fatty acidemia, (4) hyperglycemic response to glucagon [9].

CHI is a genetic disorder caused by mutations in the regulation of the potassium channel which is closely involved in insulin secretion by the pancreatic β cell. [10] The ATP-sensitive potassium channel in the cell membrane (KATP channel) has two subunits: the KIR 6.2 channel, and a regulatory component known as the sulfonylurea receptor (SUR). Inactivating mutations prevent the potassium channel from opening allowing constant inward flux of calcium and continuous insulin secretion. The genes for glucokinase and glutamate dehydrogenase can also have inactivating mutations which also close the potassium channel and result in hyperinsulinism. So far, mutations in ABCC8, KCNJ11, GLUD1, GCK, HADH, SLC16A1, HNF4A, UCP2 and HNF1A have been identified to be involved in the pathogenesis of CHI. [10–11] The most common causes of diffuse CHI are mutations in the genes ABCC8 and KCNJ11. These two genes encode for the SUR1 (sulphonylurea receptor 1 subunit) and Kir6.2 (inward-rectifying potassium channel pore-forming subunit) proteins respectively which constitute the KATP channel of the pancreatic β-cell membrane [10].

There are two histological subtypes of CHI: diffuse and focal [12]. The whole of the pancreas is affected in the diffuse form, whilst only a portion of the pancreas is involved in the focal from. The diffuse form is inherited in an autosomal recessive (or dominant) manner whereas the focal form is sporadic in inheritance. The focal lesions are localized by using a specialized positron emission tomography scan using Fluroine-18L-3, 4- dihydroxyphenyalanine isotope (18F-DOPA-PET) [13]. The early diagnosis of HH is fundamentally important for preventing hypoglycaemic brain injury hence clinicians should have a low threshold for recognizing these patients. Any patient with recurrent or persistent hypoglycaemia can potentially have CHI and this is the only cause of hypoglycaemia which persists despite continuous administration of glucose. As a result of the fetal hyperinsulinaemia, newborns with CHI may be macrosomic however; the absence of macrosomia does not exclude CHI. Hypertrophic cardiomyopathy and hepatomegaly (increased storage of glucose as glycogen) are observed in some patients with CHI. The mechanism of cardiomyopathy and hepatomegaly in these patients is unclear but might be related to the effect of fetal hyperinsulinaemia [14].

There are two parts of newborn symptoms of hypoglycemia: the decreased availability of glucose for the brain and the adrenergic stimulation. The clinical signs vary from asymptomatic hypoglycemia to severe central nervous system (CNS) and cardiopulmonary disturbances. Alterations in the levels of consciousness, unresponsiveness, lethargy, hypotonia, poor feeding, jitteriness, tremulousness, irritability, stupor, seizures, vomiting - all are part of the neonatal hypoglycemia. In addition, congestive heart failure, cyanosis, apnea, a high-pitched cry and hypothermia may be present. It is of note that the autonomic nervous system manifestations include pallor, diaphoresis, tachycardia, hunger, anxiety, nausea, and vomiting. As many neonates have additional metabolic or clinical conditions (hypoxia-ischemia, infections…) the signs of hypoglycemia are becoming even more variable. In contrast to the neonates, older children manifest mental confusion, behavioral changes, headache, amnesia, decreased visual acuity, diplopia, dysarthria, aphasia, ataxia, difficulty concentrating, somnolence, seizures, hemiplegia, paresthesias, dizziness, and coma.

A powerful clue to the dysregulated insulin secretion is the calculation of the intravenous glucose infusion rate required to maintain normoglycaemia. An intravenous glucose infusion rate of >8 mg/kg/min (normal is 4-6 mg/kg/min) is virtually diagnostic of HH [14]. In CHI there is an inappropriate concentration of serum insulin (and/or c-peptide) for the level of blood glucose (spontaneous or provoked) [15]. The metabolic effect of this inappropriate insulin secretion is reflected by the inappropriately low levels of serum ketone bodies and fatty acids during the hypoglycaemic episode. There is no correlation between the serum insulin concentration and the severity of the hypoglycaemia [16]. The Diagnostic criteria for patients with CHI is summarizes in Table 3. It is vital to make a prompt diagnosis and institute immediate management of CHI as delay in treatment may cause severe brain damage and permanent neurodevelopmental disorders. Treatment of CHI includes medical, surgical or sometimes combination therapies. The primary goal of therapy is to achieve normoglycaemia and restore ability of ketone bodies production as glucose and ketones provide main and alternative energy requirements for the brain. The clinician should focus on corrections of two metabolic disarrangements to achieve a safe normal blood glucose level (generally recommended as 463 mg/dl or 43.5 mmol/l) and to inhibit inappropriate insulin secretion [17, 18].

**Table 3:** Diagnostic criteria for patients with CHI

Glucose infusion rate >8 mg/kg/min	Serum ammonia level may be raised (HI/HA syndrome)
**Laboratory blood glucose <3 mmol/l with:** - Detectable serum insulin/C-peptide- Suppressed/low serum ketone bodies- Suppressed/low serum fatty acids	Raised plasma hydroxybutyrylcarnitine and urinary 3-hydroxyglutarate (HADH deficiency) Supportive evidence (when diagnosis is in doubt or difficult): *Positive glycaemic (>1.5 mmol/L) response to intramuscular/ intravenous glucagon *Positive glycaemic response to a subcutaneous/intravenous dose of octreotide *Low serum levels of IGFBP1 [insulin negatively regulates the expression of IGFBP1] *Suppressed branch chain (leucine, isoleucine and valine) amino acids *Provocation tests (leucine loading or exercise testing) may be needed in some patients

If the neonate is asymptomatic and able to tolerate oral/NG (nasogastric) feeds, increasing the volume and/or frequency of feeds can be tried first as long as the hypoglycemia is not severe. Oral dextrose solutions are not recommended for this purpose as they show no benefit over milk in raising glycemia. In those children with symptomatic hypoglycaemia (seizure, etc) or hypoglycaemia that is unresponsive to oral feeds, immediate treatment with an intravenous bolus of 2 ml/kg of 10% glucose should be initiated over 5 minutes. The minibolus is than followed by a constant infusion of dextrose at 6-8 mg/min/kg. [19] Alternatively, a constant infusion of at least 6-8 mg/min/kg glucose has been proven to normalize glucose concentrations 5-10 minutes later than those produced by the minibolus. While investigations are done to understand the underlying etiology, blood glucose must be kept > 65 mg/dL. It may be necessary to increase the concentration of dextrose infusion and consider central access [20]. Normal glucose requirements in a neonate are between 4 and 6 mg/kg/min, equivalent to the normal hepatic production rate of glucose. Dextrose requirements > 8 mg/kg/min suggests hyperinsulinism.

In an emergency situation where venous access is difficult to obtain, intramuscular glucagon (0.5-1 mg) can be administered in order to temporarily improve blood glucose concentrations. [14] Glucagon causes immediate release of glycogen stores from the liver and also has actions on gluconeogenesis, ketogenesis and lipolysis. It can also be administered (alone or in combination with octreotide) as an intravenous or subcutaneous infusion to stabilise blood glucose concentrations in the acute management of infants with CHI. Persistent hypoglycemia is always requiring additional investigations. There are two conditions that should be recognized and treated: fatty acid oxidation disorders and hyperinsulinism. Those conditions are often refractory to glucose replacement [21]. Diazoxide is the mainstay of medical therapy and is used as a first line drug [14] and dosage is 5- 20 mg/kg/d, administered orally. Diazoxide inhibits insulin release from β-cells by keeping KATP channels open. In diazoxide unresponsive patients, blood glucose can be stabilized using glucagon and/or octreotide along with high concentration glucose infusions Glucagon acts by releasing hepatic glycogen stores and can either be given as a bolus injection or as a continuous infusion (5-10 mcg/kg/h) to stabilize blood glucose levels.

We can also use octreotide as a somatostatin analogue that activates potassium channels in β-cell and therefore inhibits insulin release (5-30 mcg/kg/d). There is a long-acting octreotide preparation available to facilitate long-term therapy. Some cases in the literature have been reported to respond to the calcium channel antagonist Nifedipine (0.25-2.5 mg/kg/d) but in authors’ experience, it is ineffective. The indications for surgery in CHI patients include confirmed focal disease on 18F-DOPA-PET-CT scan and medically un-responsive diffuse disease. The treatment of choice for patients with the focal form of CHI is partial pancreatectomy. The exact localization of the focal lesion is made using 18F-DOPA PET-CT that helps to guide the surgeon during the surgery. The last resort for patients with medically unresponsive diffuse form of HH is a near-total pancreatectomy that unfortunately carries a high risk of exocrine pancreatic insufficiency and development of diabetes mellitus later in life. In some cases, hyperglycemia may occur within the first days after surgery, thereafter, the incidence of hyperglycemia gradually increases with age [22].

## Conclusion

Hypoglycemia continues to be an important cause of morbidity in neonates and children. Prompt diagnosis and management of the underlying hypoglycemia disorder is critical for preventing brain damage and improving outcomes. Congenital hyperinsulinism (CHI) is the most common and severe cause of persistent hypoglycemia in neonates and children. Recent discoveries of the genetic causes of CHI have improved our understanding of the pathophysiology, but its management is complex and requires the integration of clinical, biochemical, molecular, and imaging findings to establish the appropriate treatment according to the subtype.

## Competing interests

The authors declare no competing interests.
